# Safety, health, and environmental risk assessment of the aromatic outlet of Imam Khomeini Petrochemical Company using LOPA and fuzzy-LOPA and pollutants and control actions using the Bow-tie method

**DOI:** 10.3389/fpubh.2023.1251548

**Published:** 2023-10-24

**Authors:** Pariya Sarafraz, Katayoon Varshosaz, Neda Orak, Nematollah Jaafarzadeh, Ebrahim Aghajari

**Affiliations:** ^1^Department of Environmental Sciences, Islamic Azad University, Ahvaz Branch, Ahvaz, Iran; ^2^Department of Environmental Management-HSE, Islamic Azad University, Ahvaz Branch, Ahvaz, Iran; ^3^Environmental Technologies Research Center, Ahvaz Jundishapur University of Medical Sciences, Ahvaz, Iran; ^4^Department of Electrical Engineering, Islamic Azad University, Ahvaz Branch, Ahvaz, Iran

**Keywords:** risk assessment, fuzzy method, LOPA, fuzzy-LOPA, Bow-tie method

## Abstract

**Background:**

Today, with the development of the industry, the occurrence of accidents caused by the release and explosion of chemical and toxic substances in industrial units has increased, and these accidents sometimes cause irreparable damage to human life and the environment. According to a study by the American Petroleum Institute, of the recent major accidents in the last 30 years, 44% are related to machinery failure and 12% are caused by unknown factors and lack of information. Therefore, equipment risk control is aimed at preventing large and dangerous accident. The present study, the performance of LOPA and fuzzy-LOPA methods was compared toward the risk assessment of Imam Khomeini Petrochemical Company under certainty and uncertainty of data. This comparison was done in order to a conceptual method with high certainty to assess high-level hazards leading to health and safety risks and environmental pollution.

**Methods:**

First, the health, safety hazards and environmental aspects were identified via the HAZOP method. Then, a risk assessment was performed using the LOPA method. The fuzzification, severity, and likelihood of each risk were considered as an input variable and risk probability as an output variable. Finally, was the methods used in our analysis were compared and the Bow-tie software was used to draw a Bow-tie diagram to control and reduce the risks.

**Results:**

As a result, a total of 50 safety and health hazards and 37 environmental aspects were identified in the aromatic outlet of the studied company using the HAZOP method. The most critical risks identified were operational activities in feed and product tanks; flammable materials pumping; blocking the flare path; and releasing H_2_S gas. The results showed that the production of air pollutants in the power supply unit, disposal of waste from reactor tanks, disposal of waste from condensate tanks, and fire and explosion of the reactor are high-level environmental risks.

**Conclusion:**

In the conditions of uncertainty or the absence of information related to the probability and severity of the risk scenario, among the mentioned methods. The result showed that errors in the risk assessment were reduced to an acceptable extent by using Fuzzy LOPA method.

## Introduction

There are many hidden risks in today’s developed industries (1, 2). Although some of these risks cannot damage the environment, some others will have harmful effects. Therefore, it is necessary to estimate the occurrence of such hazarads as much as possible using appropriate methods. Additionally, global interest in sustainable development has increased emphasis on the social, environmental, and economic impacts of processes in industries (3, 4). So implementing new technology needs an effective and efficient risk assessment investigation to minimize the risk to an acceptable level or as low as reasonability practicable (ALARP) (5). For a reliable and correct identification of risks, a risk function can be formulated based on the severity and the likelihood of occurrence (6, 7). Hazard identification, qualitative risk analysis, elimination, modification, control, and monitoring of risks are among the most important, necessary, and technical needs of safety and process engineers of all industries. After identifying the hazards and determining their attributed quality, the hazards with medium and high-risk are evaluated in terms of quantitative risk assessment criteria (8–10). Nowadays, in the design of process units, quantitative risk assessment and consequence modeling must be performed along with the design of protective layers against catastrophic events before or during the event. Without conducting such assessments, an acceptable margin of safety cannot be achieved for the operating units in terms of environmental aspect (11, 12). However, the disadvantage of the methods used in the quantitative risk assessment method is the data imperfection and the lack of robustness in the results (13). Moreover, fuzzy logic methods can be used to collect and process the information and the data and solve the problems related to inaccuracy (14). Identifying and rating risk factors is very important in the decision-making process as well as the risk assessment. Layer of protection analysis (LOPA) and fuzzy logic are used to identify the safety integrity level (SIL) required for safety-critical functions in the industry. Therefore, it can be concluded that LOPA can be an efficient way to obtain quantified results and determine whether the system’s existing independent protection layers (IPL) are sufficient. Finally, the fuzzy logic method is used to determine the severity and the SIL rate. Fuzzy logic is integrated to deal with the uncertainty of real-world conditions (15–17). Due to the increasing possibility of irrecoverable environmental, health, and safety risks in petrochemical industries, such research seems to be necessary (18). Several studies have been conducted in the field of hazard identification, but so far, no study has been conducted in the field of environmental, health and safety risk assessment of equipment in the petrochemical industries using the Fuzzy LOPA method. This research was performed in the aromatic outlet of Imam Khomeini Petrochemical Company to evaluate the safety, health, and environmental risks of the equipment. The risks were evaluated after identifying them using the hazard and operability study (HAZOP) method. To solve the issue of the inaccuracy of the data, the severity and likelihood of occurrence of risk were calculated using the fuzzy method and displayed with a Bow-tie diagram. The methods were then compared under data certainty and uncertainty to determine the efficient method.

The main aim of this study is compared toward the risk assessment of aromatic outlet Imam Khomeini Petrochemical Company under certainty and uncertainty of data. Sub-goals in this study are Identifying and classifying the hazards of the aromatic outlet equipment, risk assessment of aromatic outlet equipment in the certainly and uncertainly information, evaluation and analysis of independent protective layers used to reduce the risk of aromatic outlet equipment, actions to control, reduce and eliminate possible risks. These goals are used in petrochemicals, chemical industries, for students and researchers and educational centers.

This research answers these questions: What are the safety and health hazards in the aromatic outlet based on the HAZOP method? What are the environmental aspects in aromatic outlet based on the HAZOP method? What are the levels of safety, health and environmental risks in aromatic outlet on LOPA method? What are the major scenarios of safety, health and environmental based on the Bow-tie method? Does the fuzzification of the LOPA risk assessment method reduce the uncertainty in the results?

Use quantitative risk assessment studies with the design of protective layers against catastrophic events such as LOPA is one of the requirements for the design of manufacturing units, and without conducting such studies, cannot expect an acceptable safety level for industries. As a result, LOPA creates a logical framework for allocating resources and controlling risks. But sometimes, limitations such as the unavailability or uncertainly of information, reduce the efficiency of risk assessment methods, which is solved by fuzzifying this problem, and due to the complexity of petrochemical units and the graphic representation of Bow-tie method, it is showed a simple understanding of the risk assessment process.

In the world until today, many studies have been done in the field of hazard identification and risk assessment, but the research in the field of aromatic unit equipment risk assessment hasnot been conducted using the LOPA method and Bow-tie software for easy and quick understanding. Also, use and determination of the conceptual model and comparative research in the certainly and uncertainly of information has not been investigated. Therefore, such study is necessary.

## Materials and methods

The statistical population of the present study was the aromatic outlet of Imam Khomeini Petrochemical Company in Mahshahr, Iran, and the statistical samples were all the equipment of this complex. The degree of importance of the risk was determined after identifying the factors causing it using HAZOP. Scenarios were extracted based on the priority of the risks. The scenarios of safety, health, and environment were evaluated using simple LOPA and Bow-tie methods. Finally, the results of risk assessment in the LOPA method were fuzzified using Matlab, and the results were compared afterwards. In this research, we followed the four steps below:

In the first step, the equation of severity and the likelihood of occurrence were used to determine the level for the identified hazards. In HAZOP First, a detailed description of the process is prepared, then the system is divided into smaller study units and questions are systematically asked in each study unit to determine what deviations and how they may occur and whether these deviations lead to danger or not. The likelihood of occurrence was ranked from 1 to 6 (the higher garde was attributed to the higher probability), and the severity was ranked from 1 to 9 (the higher grade was attributed to the higher level of severity). Eventually, the classification of hazards included three categories of high, medium, and low (19).

In the second step, LOPA was performed using an adverse consequence of the results of the HAZOP analysis. LOPA can be considered as a simple form of quantitative risk assessment. This technique focuses on reducing hazards by determining independent protection layers against an incident scenario. Then the severity of this consequence was estimated. The initial causes were then identified for each consequence. IPL and probability of failure on demand (PFD) were determined for each of the initial causes. Finally, based on the results, the risk was reduced by adding more IPL or re-designing the process. In this method, the likelihood of occurrence was 1 to 7 (the higher grade was attributed to the higher likelihood of occurrence). The severity was ranked from 1 to 7 (the higher grade was attributed to the higher severity) (20, 21).

**Figure 1:**
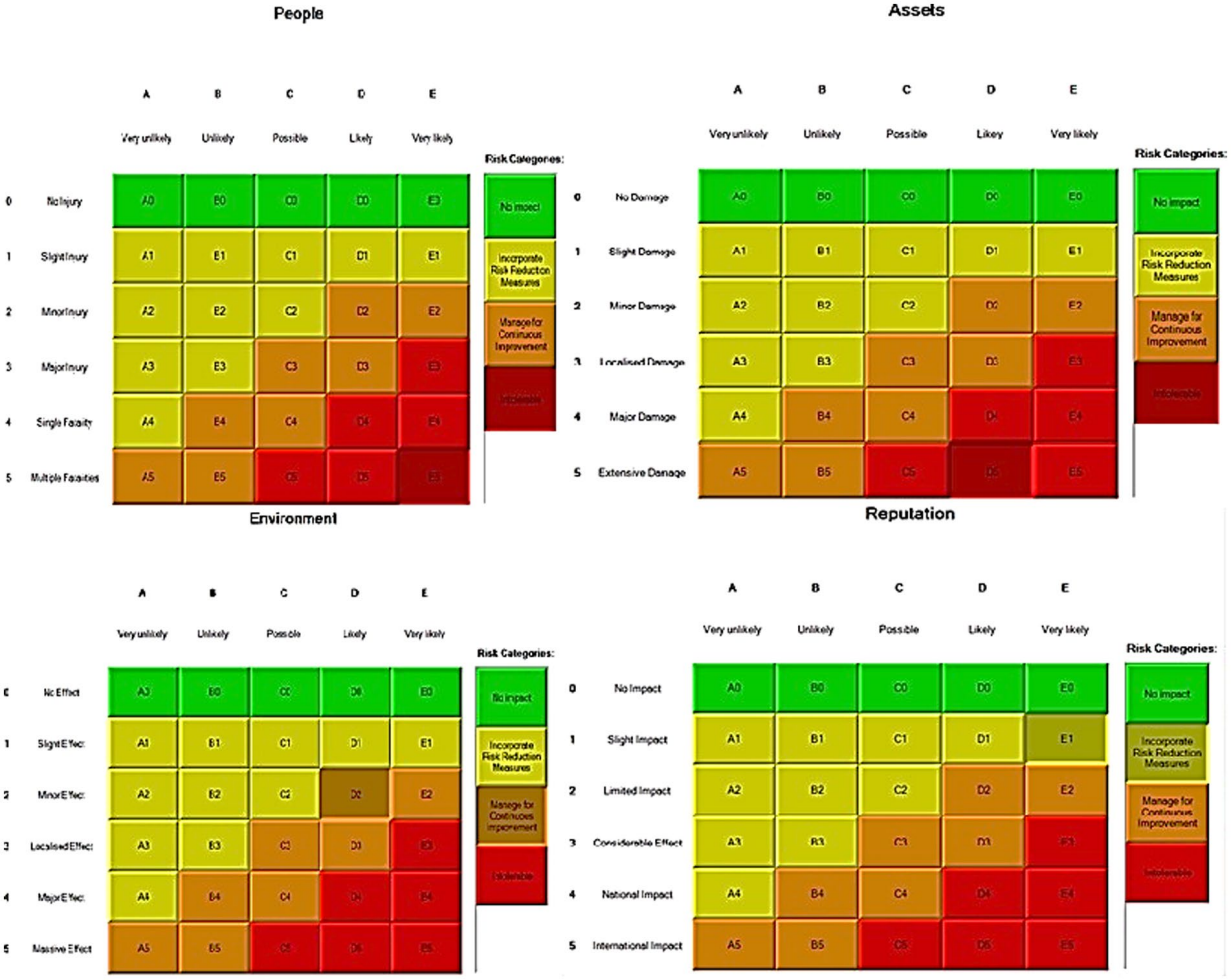
Graphical presentation of the environmental scenario by the Bow-tie method – Scenario 1.

In the third step, after assessing the scenarios using the LOPA method, the Bow-tie method was used to graphically display the scenarios. Bow-tie xp software was used to make the Bow-tie diagram and assess the scenarios. This software is for risk assessment and analysis. Also, It can use this software for validation certainty and uncertainty by comparing with a series of information from the past. First, the location was determined in center of diagram (red circle), second, the hazard and important incident were determined in top of circle (yellow box). Third, threats were added in left of diagram (blue boxes). Fourth, the consequences were determined in right of diagram. Then, control ways were determined before consequences (white boxes). Aggravating factors (if any) were added, and finally, the consequences were assessed based on the severity of 0–5 and the likelihood of occurrence of A–E in terms of the credibility of the organization (Khomeini Petrochemical Company), environment (water, soil and resources of petrochemical Imam Khomeini and neighboring areas of industry), equipment (equipment of aromatic unit), and people (Industry workers). Use code beneath the red boxes is according to Figure 1 below (22).

In the fourth step, Matlab 2013a was used to perform the fuzzification process. Fuzzy is one of the subsets of artificial intelligence and is used as a model for using uncertainly information. It is based on the principle that the degree of membership of an element to a set can change from zero to one. The fuzzy command was executed using the fuzzy toolbox (23). First, linguistic variables were determined for each of the inputs and outputs. The fuzzification section converts absolute (non-fuzzy) input variables [severity, frequency, and system output (risk level)] into fuzzy numbers. In the present study, the triangular membership function was used. The second step was a fuzzy inference, which converts input fuzzy sets into output fuzzy sets. Here, the max-min inference method was used to obtain the fuzzy output. Fuzzy rules were defined based on the number of functions of each of the input variables. The last step was the de-fuzzification section. In this step, the weighting process was performed and a non-fuzzy output number was presented. One of the most famous de-fuzzifier is the center of gravity de-fuzzifier. Then the created fuzzy relation section was presented in the view menu. Finally, to assess the efficiency of the fuzzy-LOPA model, the results were compared to the classic LOPA method.

In the table below, showed variation the ranges chosen for like hood and severity in LOPA and fuzzy methods (Table 1).

**Table 1:** Ranges choose for frequency and severity in LOPA and Fuzzy LOPA.

Linguistic variables		Fuzzy sets	Description range	Fuzzy	X
Input	Frequency (F)	Very high	$F^{\in }$ [10^−2^, 1) [1/year]	−0.2, 0, 0.1	$X_{F}^{\in }$ (10^-8^,10^0^)
		High	$F^{\in }$ [10^−1^, 10^−3^)	0.05, 0.17, 0.0.3	
		Moderate	$F^{\in }$ [10^−2^, 10^−4^)	0.2, 0.35, 0.45	
		Low	$F^{\in }$ [10^−3^, 10^−5^)	0.4, 0.55, 0.65	
		Very low	$F^{\in }$ [10^−4^, 10^−6^)	0.6, 0.75, 0.8	
		Unlikely	$F^{\in }$ [10^−5^, 10^−7^)	0.75, 0.85, 0.95	
		Remote	F < 10^−6^	0.9, 1, 1.2	
	Severity (S)	Negligible	$S^{\in }$ [1,2)	−0.4, 0, 0.2	$X_{S}^{\in }[$ 1,5)
		Low	$S^{\in }$ [2,3)	0.1, 0.25, 0.4	
		Moderate	$S^{\in }$ [3,4)	0.3, 0.45, 0.6	
		High	$S^{\in }$ [4,5)	0.5, 0.65, 0.85	
		Very high	$S^{\in }$ [5)	0.75, 1, 1.2	
Output	Risk category (R)	A: Acceptable	$R^{\in }$ [0,2)	−0.2, 0, 0.2	$X_{R}^{\in }[$ 0,5)
		TA: Tolerable Acceptable	$S^{\in }$ [1,3)	0.1, 0.3, 0.5	
		TNA: Tolerable Unacceptable	$S^{\in }$ [2,4)	0.4, 0.6, 0.8	
		NA: Unacceptable	$S^{\in }$ [3,5)	0.7, 1, 1.2	

## Results

At first for compared LOPA and Fuzzy LOPA, a total of 50 safety and health hazards and 37 environmental aspects were identified in the aromatic outlet of Imam Khomeini Petrochemical Company using the HAZOP method. Among the identified hazards, 17, 19, and 14 risks were at a low, medium, and high level, respectively. The most critical health and safety risks identified were operational activities in the area of feed and product tanks, pumping of flammable materials, blocking the path to the flare, and releasing H_2_S gas. The results showed that the production of air pollutants in the power supply unit, disposal of waste from the reactor tanks, disposal of waste from the condensate tanks, and fire and explosion of the reactor were high-level environmental risks. From the 50 studied health and safety hazards and 37 environmental aspects, 28 and 12 cases were extracted to create scenarios, respectively.

Health and safety scenarios are chemical spraying due to leakage from transfer lines, chemical fire in the operation area, the explosion of tanks in the operation area, chemical leakage caused by Ethylene Dichloride (EDC) injection, fire and explosion caused by pumping flammable materials, fire and explosion caused by the operation/stopping unit start-up in emergency situations, fire and explosion caused by stopping unit start-up in emergency situations, spraying materials in the process of injecting materials (Javel water injection), blockage of the excess gas path toward flare and explosion, the emission of infrared waves caused by the activity of unit furnaces, noise pollution caused by compressors, contact with H_2_S gas when entering the reactor, contact of vapors with the skin during repairs, contact with aromatic substances during routine operations, fire and electric shock during repairs in substation, leakage of H_2_S gas in area 100, leakage and inhalation of argon gas by people in the control room, fall from height during working on scaffolding, respiratory problems in confined conditions, spillage of chemicals during unloading and loading of solvent tanks, inhalation of dust during the unloading and loading of soil to the DA-401 column, fire during the use of nitrogen gas contaminated with carbon hydrate to purge fire equipment, the release of H_2_S gas due to disruption in the flow of reactor effluent disposal, release of H_2_S due to disruption of recycle gas flow to the stripper, release of H_2_S due to sour water flash drum, leakage of toxic gases due to disruption in the hydrodesulfurization (HDS) recycling compressor, leakage of H_2_S gas due to disruption of sulfiding flow in HDS, disruption in the gas absorber in the reactor.

Environmental scenarios are discharge of wastewater during wastewater treatment, the emission of gases from the combustion of furnaces, discharge of wastewater during cooling column operation (4 and 6), the emission of gases from the burning of gases in the torch, increasing the seawater return temperature, condensation operation of the exhaust steam from the GT-201 aromatic unit turbine, discharge of water from washing the operating environment, release of hydrocarbon vapors due to rising water temperature in corrugated plate interceptor (CPI), release of catalyst in the environment during the operation of catalyst discharge and replacement, the production of air pollutants caused by the electricity supply of the unit, effluent reactor wastewater disposal, wastewater overflow from condensate tanks, fire and explosion of the reactor.

The analysis of safety and health scenarios using the LOPA method indicated that 6 scenarios were at the acceptable level without revision, 8 scenarios were at the acceptable level with revision, 10 scenarios were at the unacceptable level with medium priority and 4 scenarios were at the unacceptable level with immediate priority. Also, as far as the environmental scenarios, 4 scenarios were at the acceptable level without revision, 1 scenario was at the acceptable level with revision, 3 scenarios were at the unacceptable level with medium priority, and 4 scenarios were at the unacceptable level with immediate priority. Moreover, for environmental scenarios, 4 scenarios were at the acceptable level without revision, 1 scenario was at the acceptable level with revision, 3 scenarios were at the unacceptable level with medium priority, and 4 scenarios were at the unacceptable level with immediate priority.

Although these scenarios had the lowest score in frequency of occurrence, their extreme severity requires strong safety management. Generally, in any organization, emergencies that lead to major incidents must be managed immediately.

**Figure 2:**
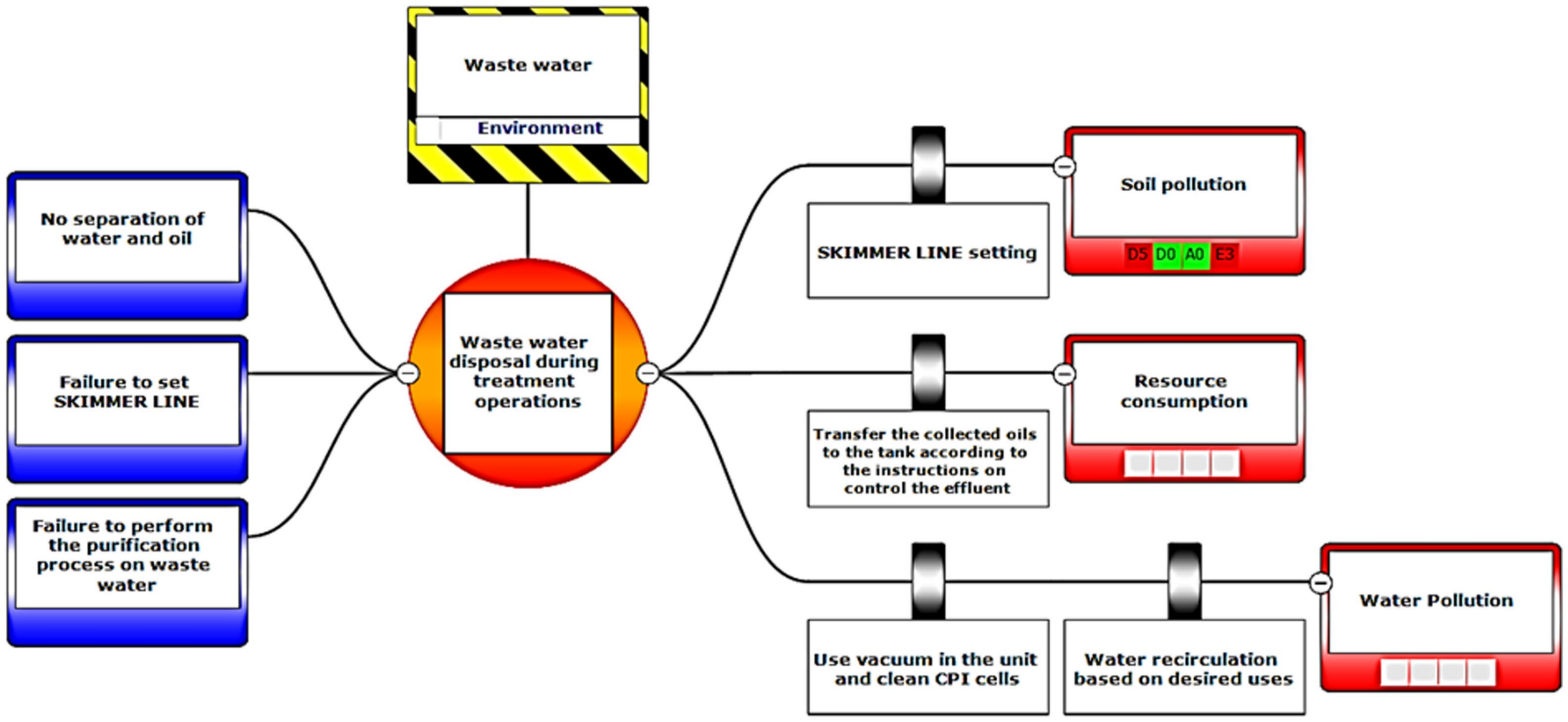
Graphical presentation of the environmental scenario by the Bow-tie method – Scenario 2.

**Figure 3:**
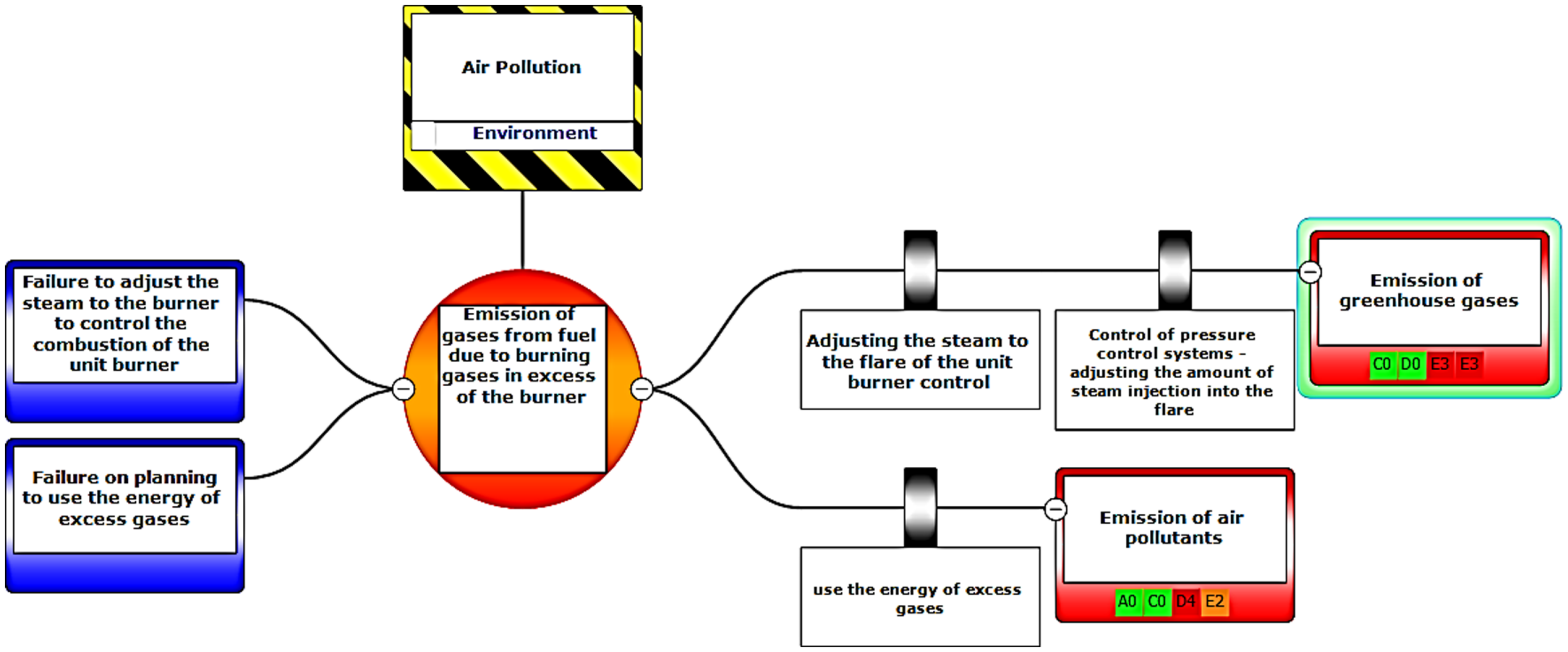
Graphic representation of the environmental scenario by the Bow-tie method – Scenario 9.

**Figure 4:**
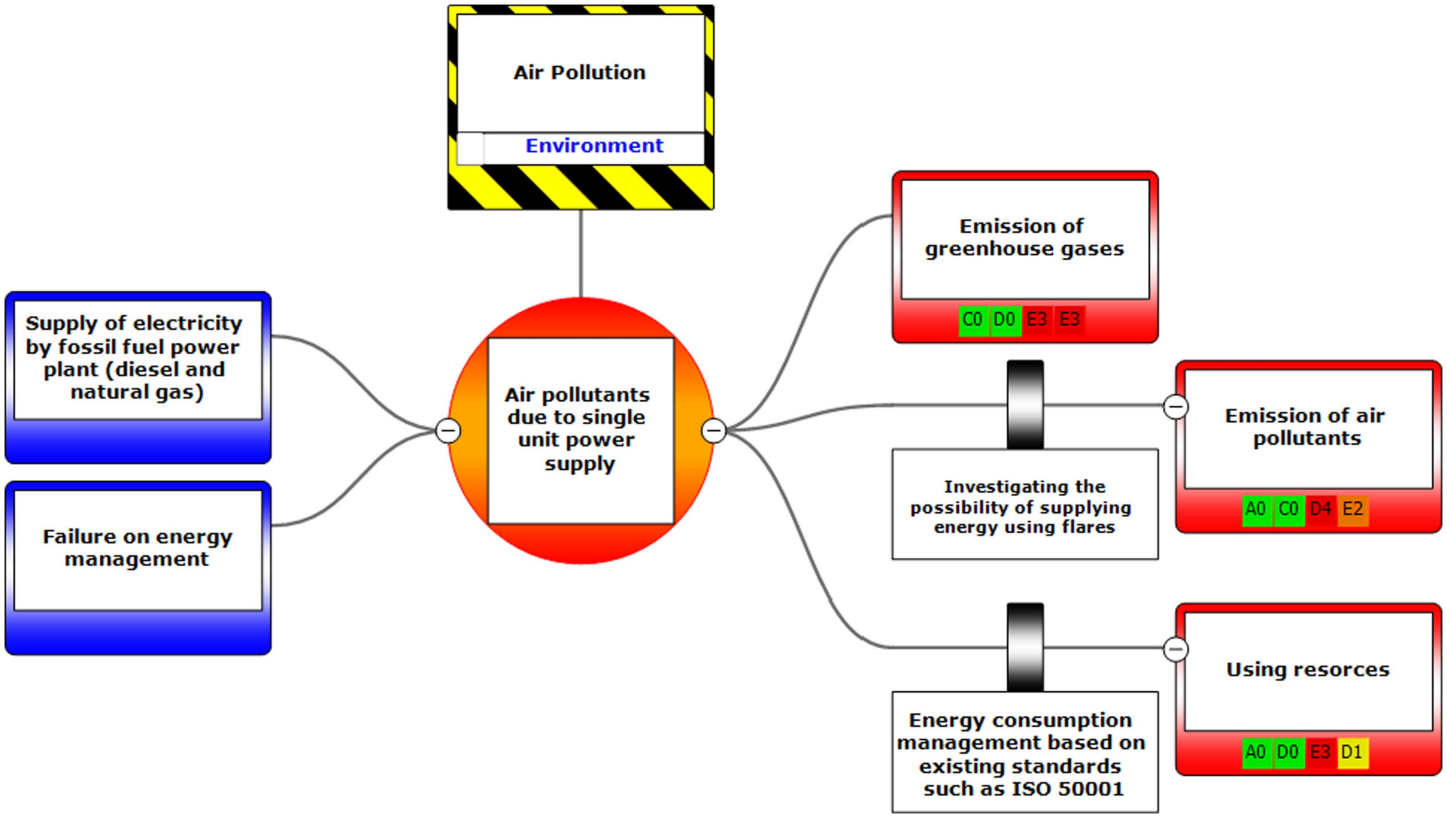
Graphic representation of the environmental scenario by using the Bow-tie method – Scenario 12.

**Figure 5:**
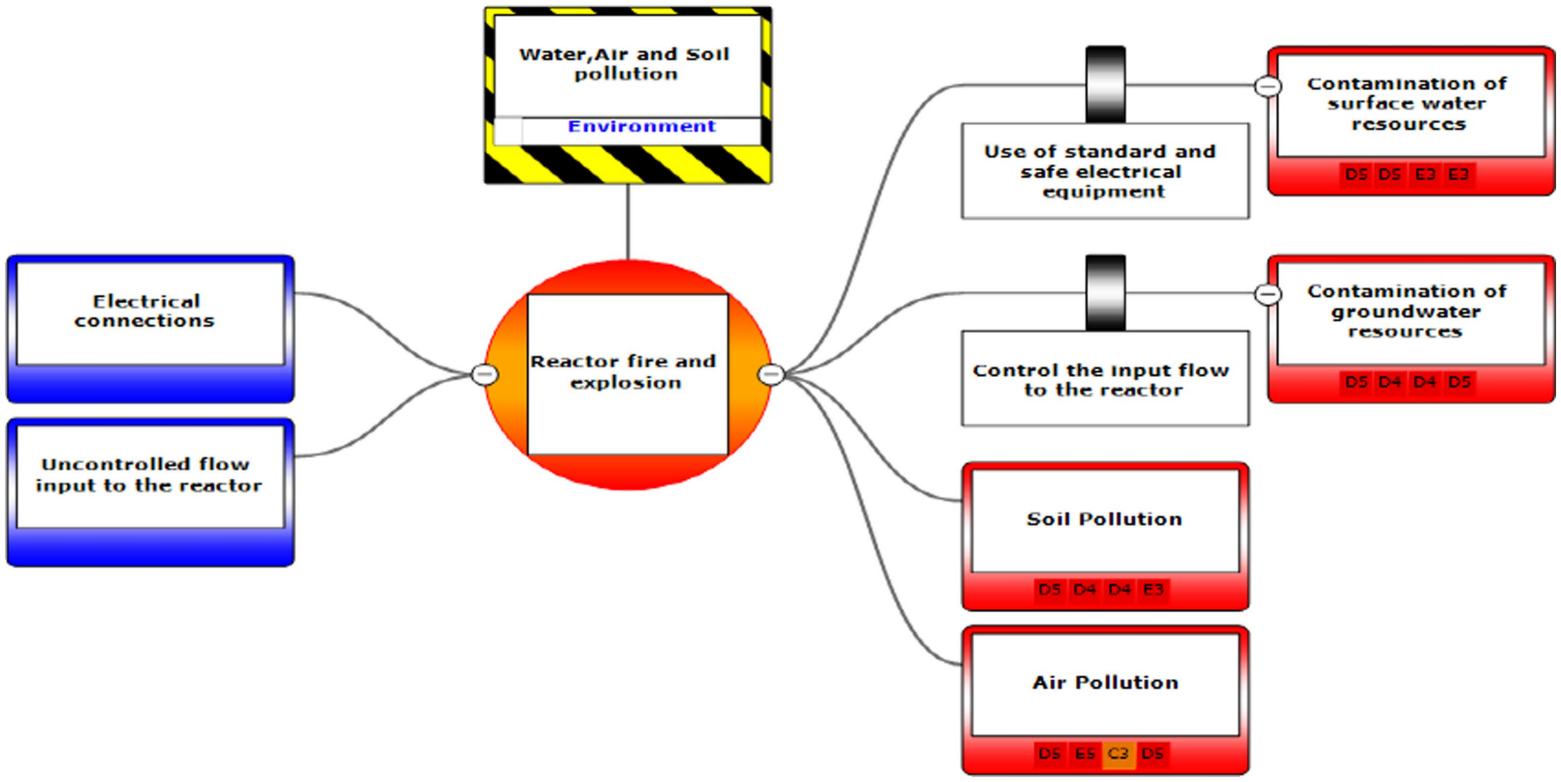
Graphic representation of the health and safety scenario by using the Bow-tie method – Scenario 23.

Eventually, Bow-tie software was used to present methods of controlling environmental pollutants as well as the health and safety risks. The bow-tie diagrams are presented below in Figures 2–5 to show high environmental risks.

**Figure 6:**
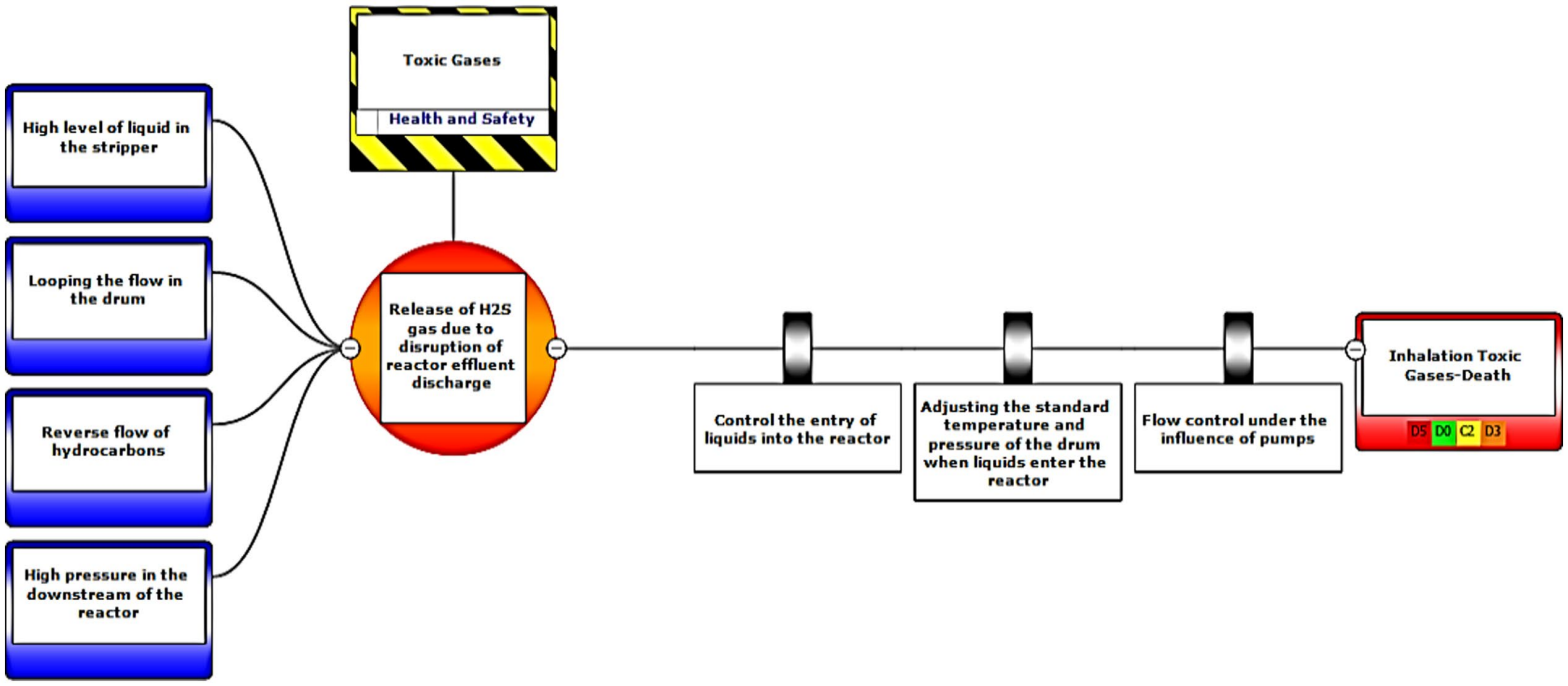
Graphic representation of the health and safety scenario by using the Bow-tie method – Scenario 6.

**Figure 7:**
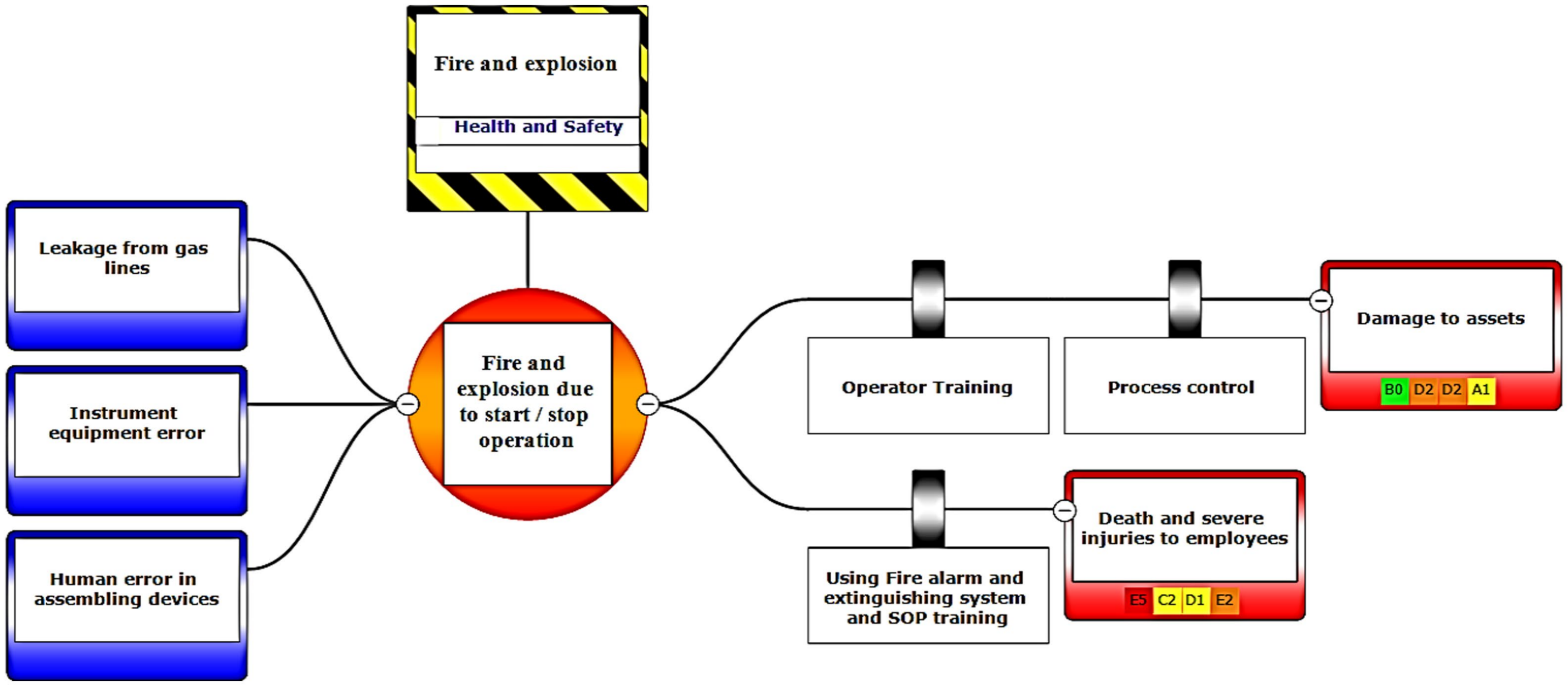
Graphic representation of the health and safety scenario by using the Bow-tie method – Scenario 12.

**Figure 8:**
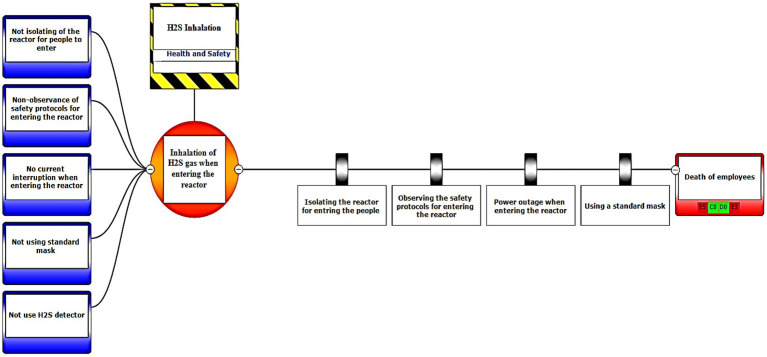
Graphic representation of the health and safety scenario by using the Bow-tie method – Scenario 14.

**Figure 9:**
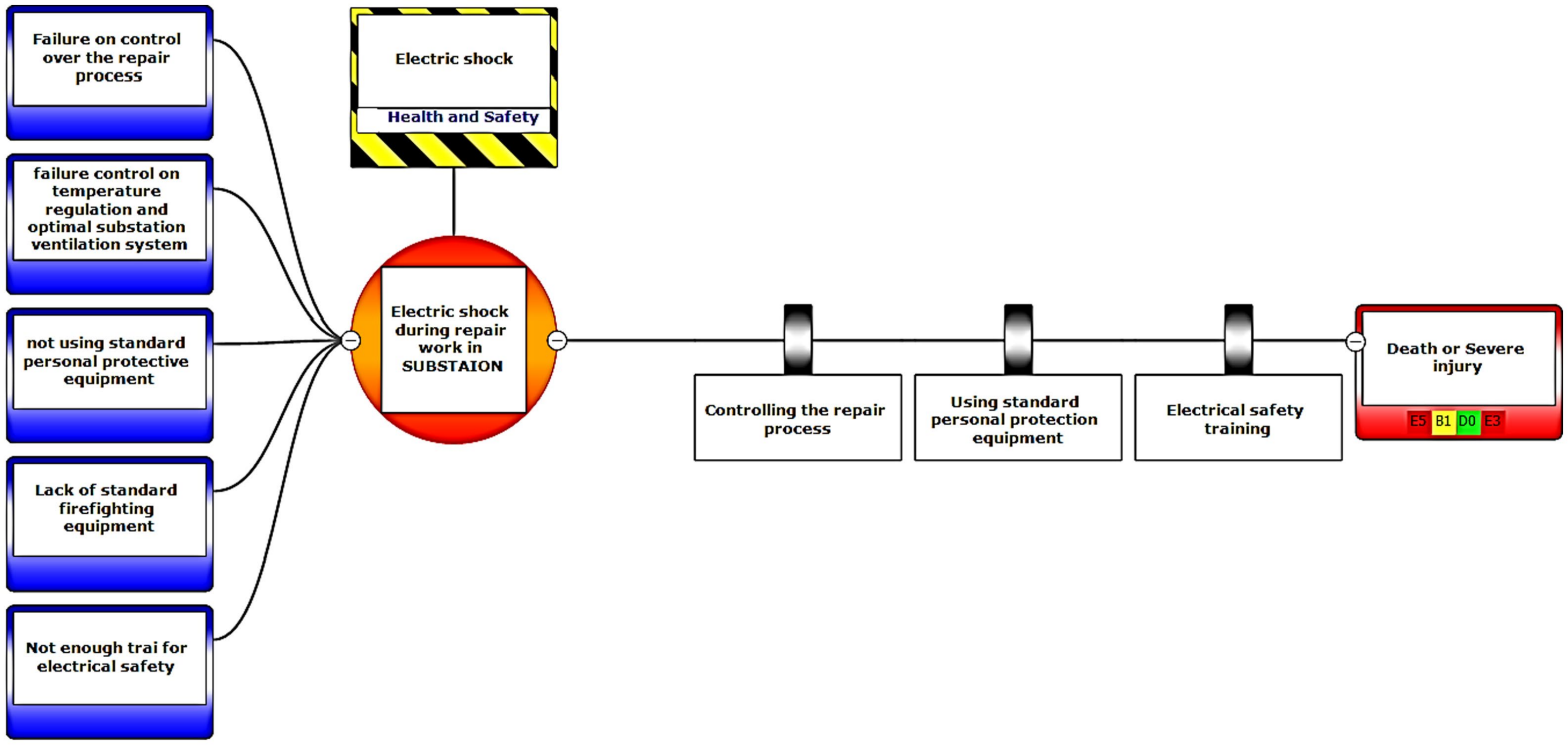
Graphic representation of the health and safety scenario by using the Bow-tie method – Scenario 16.

**Figure 10:**
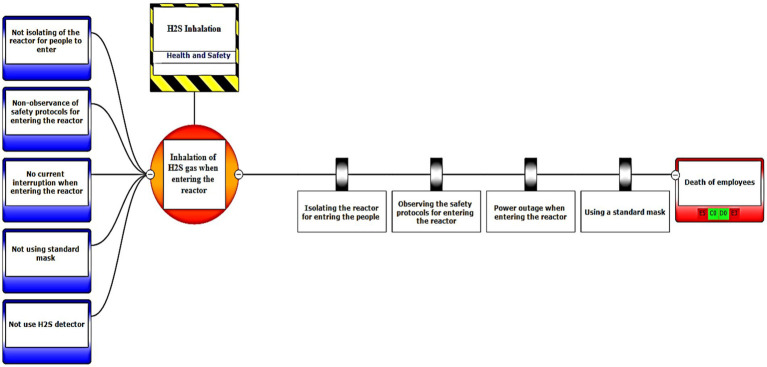
Risk assessment matrixs for in Bow-tie method.

The bow-tie diagrams are presented below in Figures 6–10 to show high health and safety risks.

In the diagrams presented in above figures, 4 environmental scenarios and 5 health safety scenarios with high risk were investigated. The error tree is drawn on the left side and the event tree is on the right side, and the risk is placed in the center of the diagram as a node, and finally the decision was made at 4 levels: the organization, environment, equipment, and people. For an example in scenario 1, the scenario is waste water disposal during treatment operations. The location was aromatic outlet, second, the hazard and important incident were noseparation of water and oil, failure to set skimmer line and failure to perform the purification process on waste water. Third, threats were soil pollutions, resource consumption and water pollution. Fourth, the consequences were determined. Then, control ways were skimmer line setting, transfer the collected oils to instructions on control of effluent, use vacuum in the unit and clean cpi cells, water recirculations based on desired uses, and finally, determine the levels risk of the credibility of the organization in aromatic outlet of Imam Khomeini Petrochemical Company is E3 (intolerable), environment D5 (intolerable), equipment of oromatic outlet D0 (no impact), and people in aromatic outlet A0 (no impact).

According to the main purpose of the research, risk assessment results using the LOPA method in the conditions of certainty and its fuzzification in the conditions of data uncertainty or lack of information showed that the LOPA fuzzy method is highly reliable with a high certainty method and with Bow-tie diagram, each of the elements and their relationship with this collection can be clearly seen in the whole collection, which helps the process of reviewing the process, and by using it, will be able to quickly, easily and clearly see your results (Figures 3–9).

## Discussion

The results were compared with the classic LOPA method in the Tables 2, 3 to assess the efficiency of the fuzzy-LOPA model. Table 4 shows the abbreviations used in Tables 2, 3.

**Table 4:** The terms used in LOPA & Fuzzy-LOPA.

L:LOW
M: MEDIUM
H: HIGH
A: ACCEPTABLE
TA:TOLERABLE ACCEPTABLE
U: UNACCEPTABLE
TU:TOLERABLE UNACCPETBELE

**Table 2:** Comparison of the results of environmental scenarios assessment using LOPA and fuzzy-LOPA methods in the aromatic unit.

Row	Scenarios	Fuzzy LOPA	LOPA	HAZOP
1	Discharge of wastewater during wastewater treatment	U	Unacceptable-immediate priority (H)	M
2	The emission of gases from the combustion of furnaces	TU	Unacceptable-medium priority (M)	M
3	Discharge of wastewater during cooling column operation (4 and 6)	TA	Acceptable with revision (L)	M
4	The emission of gases from the burning of gases in the torch	U	Unacceptable-immediate priority (H)	M
5	Increasing the seawater return temperature Condensation operation of the exhaust steam from the GT-201 aromatic unit turbine	A	Acceptable without revision (L)	M
6	Discharge of water from washing the operating environment	A	Acceptable without revision (L)	M
7	Release of hydrocarbon vapors due to rising water temperature in (CPI)	A	Acceptable without revision (L)	M
8	Release of catalyst in the environment during the operation of catalyst discharge and replacement	A	Acceptable without revision (L)	M
9	The production of air pollutants caused by the electricity supply of the unit	U	Unacceptable-immediate priority (H)	H
10	Effluent reactor wastewater disposal	TU	Unacceptable-immediate priority (H)	M
11	Wastewater overflow from condensate tanks	TU	Unacceptable-immediate priority (H)	H
12	Fire and explosion of the reactor	U	Unacceptable-immediate priority (H)	H

**Table 3:** Comparison of the results of health and safety scenarios assessment using HAZOP, LOPA, and fuzzy-LOPA methods in the aromatic unit.

Row	Scenarios	Fuzzy LOPA	LOPA	HAZOP
1	Chemical spraying due to leakage from transfer lines	TA	Unacceptable-medium priority (M)	M
2	Chemical fire in the operation area	TU	Unacceptable-medium priority (M)	M
3	The explosion of tanks in the operation area	TU-TA	Unacceptable-medium priority (M)	H
4	Chemical leakage caused by EDC injection	TA	Acceptable with revision (L)	M
5	Fire and explosion caused by pumping flammable materials	TU	Unacceptable-medium priority (M)	H
6	Fire and explosion caused by the operation/stopping unit start-up in emergency situations	U	Unacceptable-immediate priority (H)	H
7	Fire and explosion caused by stopping unit start-up in emergency situations	TA	Acceptable with revision (L)	M
8	Spraying materials in the process of injecting materials (Javel water injection)	TA	Acceptable with revision (L)	M
9	Blockage of the excess gas path toward flare and explosion	A	Acceptable without revision (L)	H
10	The emission of infrared waves caused by the activity of unit furnaces	A	Acceptable without revision (L)	M
11	Noise pollution caused by compressors	TA	Acceptable with revision (L)	M
12	Contact with H_2_S gas when entering the reactor	TU	Unacceptable-medium priority (M)	H
13	Contact of vapors with the skin during repairs	A	Acceptable without revision (L)	M
14	Contact with aromatic substances during routine operations	TA	Acceptable with revision (L)	M
15	Fire and electric shock during repairs in substation	U	Unacceptable-immediate priority (H)	H
16	Leakage of H_2_S gas in area 100	U	Unacceptable-immediate priority (H)	H
17	Leakage and inhalation of argon gas by people in the control room	TA	Acceptable with revision (L)	M
18	Fall from height during working on scaffolding	TU	Unacceptable-medium priority (M)	H
19	Respiratory problems in confined conditions	TU	Unacceptable-medium priority (M)	H
20	Spillage of chemicals during unloading and loading of solvent tanks	TA	Acceptable with revision (L)	M
21	Inhalation of dust during the unloading and loading of soil to the DA-401 column	A	Acceptable without revision (L)	M
22	Fire during the use of nitrogen gas contaminated with carbon hydrate to purge fire equipment	A	Acceptable without revision (L)	M
23	The release of H_2_S gas due to disruption in the flow of reactor effluent disposal	U	Unacceptable-immediate priority (H)	H
24	Release of H_2_S due to disruption of recycle gas flow to the stripper	TU	Unacceptable-medium priority (M)	H
25	Release of H_2_S due to sour water flash drum	TU	Unacceptable-medium priority (M)	H
26	Leakage of toxic gases due to disruption in the HDS recycling compressor	A	Acceptable without revision (L)	M
27	Leakage of H_2_S gas due to disruption of sulfiding flow in HDS	TA	Acceptable with revision (L)	H
28	Disruption in the gas absorber in the reactor	TU	Unacceptable-medium priority (M)	H

Wang et al. (24) showed that by using the HAZOP-LOPA-SIL method for a safety assessment on the production process, the risk matrix and the risk measure become tolerable and appropriate to the safety level of the industry. Dass and Innal reported that by installing an effective safety barrier, industry accidents can be greatly reduced and the uncertainty problem is solved with fuzzy logic, helping decision makers to understand how they can take actions to reduce the risks associated with Liquefied Petroleum Gas (LPG) storage (25). Iddir demonstrated that evolution of the LOPA method into a fully quantitative method with a fuzzy method involves increasing the value of decision making with high confidence (26).

The analysis of environmental scenarios indicated that the most important environmental aspect in Imam Khomeini Petrochemical Company’s aromatic outlet is wastewater discharge during the sewage treatment. Failure to separate water and oil, failure to adjust the skimmer line, and failure to treat wastewater are among the reasons leading to the occurrence of this aspect. Other important environmental aspects of this unit that require environmental management include the emission of gases from the burning of gases in the torch and the pollution caused by the activity of steam boilers used for generating the electrical energy. Irvan et al. (27) reported that the emission of toxic gases is the most important environmental aspect of the petrochemical industries.

The most important identified hazard caused by the equipment in the aromatic unit was the release of H_2_S gas with 6 different scenarios. High doses of H_2_S are extremely lethal, and low doses are toxic and carcinogenic. Failure to strictly follow safety protocols in reactors and tanks, no use of identifiers, poor isolation of the equipment and lines, and an increase in the pressure of flow lines are among the factors that contributed to this scenario.

Fire and electric shocks during repairs are other important health and safety scenarios. Poor internal coordination and the non-use of personal protective equipment are two important factors in the occurrence of accidents caused by electric shock. Since high-voltage electricity is used in the complex, these accidents can cause death or severe injury. Reactor explosion, which is often caused by electrical connections or leakage of oil derivatives, is another accident scenario.

Equipment failure in the aromatic industry unit contributes a lot to major accidents and can have harmful financial, economic and environmental effects. On the other hand, due to the complexity of the equipment and the lack of detailed information, by using methods beyond the classical method, accurate and reliable risk assessment can be achieved.

According to the comparative Tables 2, 3, the risk class obtained in the presence of information and the absence of information has a significant similarity, and this indicates the good performance of the conceptual model obtained by the Fuzzy LOPA method. Finally, for a simple and comprehensive understanding, displaying high risks graphically creates a clear and comfortable understanding. According to the obtained results, the combination of these methods together is an efficient model for assessing the risk of different sectors of the industry.

One of the limitations of this research is determining the scope of its environmental consequences according to their nature.

Executive proposals of the research included to evaluate the compliance status of the design and installation of all aromatic equipment with American Petroleum Institute (API) standards. The warning systems for the release of toxic and dangerous gases and fire should be reviewed at monthly intervals. Update the work permit issuing system, especially for installation activities. Update the theoretical and practical health and safety training courses for employees of the aromatic department. The supply chain system be updated to identify defective and non-standard equipment and parts entering.

Research proposals included that some scenarios are identified and evaluated using mathematical models such as Aloha and Phast. It is suggested that the impact of the identified environmental scenarios on the water and air environment be investigated and researched further.

## Conclusion

According to main aim, compared toward the risk assessment of aromatic outlet Imam Khomeini Petrochemical Company under certainty and uncertainty of data and use suitable conceptual model with Fuzzy LOPA and Bowtie. This study showed that the level of the risks determined in LOPA under the condition of no available information is similar to the risk obtained under the condition of available information. This indicates that the results of the fuzzy-LOPA method in systems where the information is not available or the system is complex have higher certainty.

## Data availability statement

The original contributions presented in the study are included in the article/supplementary material, further inquiries can be directed to the corresponding author.

## Author contributions

All authors listed have made a substantial, direct, and intellectual contribution to the work and approved it for publication.
